# Resilience in female athletes: a meta-analysis of levels, gender differences, and associations across psychosocial, performance, and health domains

**DOI:** 10.3389/fpsyg.2026.1948554

**Published:** 2026-09-03

**Authors:** Xiangnan Li, Jiajie Pan, Rouhan Liu, Yao Chen, Xinxue Zhu, Yongqiang Su

**Affiliations:** 1 Center for Mental Health Education, Shaoxing University, Shaoxing, China; 2 Center for Positive Psychological Experiences, Shaoxing University, Shaoxing, China; 3 Department of Psychology, Shaoxing University, Shaoxing, Zhejiang, China; 4 Center for Brain, Mind and Education, Shaoxing University, Shaoxing, Zhejiang, China

**Keywords:** female athletes, gender differences, mental health, mental toughness, psychological resilience

## Abstract

**Introduction:**

Psychological resilience is considered a central component of athletes adaptability, competitive performance, and psychosocial functioning. However, the evidence regarding psychological resilience among female athletes remains fragmented, particularly with regard to its levels, gender differences, and associated psychological factors.

**Methods:**

This meta-analysis followed the PRISMA guidelines and synthesized 76 studies involving 27,386 coded participants, including at least 14,804 female athletes and 9,719 male athletes with sex-specific sample sizes available. This study examined resilience-related levels among female athletes, gender differences, and female-specific associations across psychosocial, performance, and health-related domains.

**Results:**

The results indicate that female athletes resilience-related scores were generally located above the theoretical midpoints of the measures used, although comparative studies showed that female athletes scores were slightly lower than those of male athletes. Formal moderator tests supported age stage, but not construct type, competitive level, sport type, or injury/adversity context, as a source of variation in female-male differences. Correlational analyses indicated that higher resilience-related capacities were associated with more favorable psychosocial indicators, better coping and adaptation, lower distress or risk indicators, and stronger performance- or fitness-related indicators.

**Discussion:**

The findings suggest that resilience in female athletes is best understood as a situational resource for athletic performance and health, rather than an unconditional psychological advantage. These findings are consistent with relational and resource based models of resilience and suggest that future efforts to support female athletes should combine the development of psychological capacities with improvements in the supportive environments in which they train and compete.

## Introduction

Competitive sport places athletes in repeated situations in which performance and psychological health are tested at the same time. Athletes are evaluated publicly, selected and deselected (Arnold and Fletcher, 2012), exposed to intensive training loads (Reardon et al., 2019), and required to manage injury, recovery uncertainty, and repeated success-failure feedback (Fletcher and Sarkar, 2012). In women’s sport, these demands occur alongside conditions that are not evenly distributed across sport populations, including gender stereotypes about competence (Chalabaev et al., 2013), body monitoring and psychosocial support needs in young female athletes (McManama et al., 2021), menstrual and endocrine health concerns and relative energy deficiency in sport (Mountjoy et al., 2018), and harassment risk and mental health support needs in sport systems (Reardon et al., 2019). Psychological resilience offers a useful framework for this problem because it concerns adaptation under stress, whether adaptation is indexed by performance, mental health, injury recovery, help-seeking, or continued participation. Therefore, it is necessary to further explore female athletes’ resilience-related levels and their associations across psychosocial, performance, and health-related domains.

Although there has been growing focus on athletes’ resilience, previous studies have used different definitions and measurement methods for this concept. Psychological resilience is often defined as positive adaptation in the context of adversity, risk, or stress (Luthar et al., 2000; Masten, 2001), yet contemporary models caution against treating resilience as a fixed attribute that has the same meaning in every setting (Denckla et al., 2020). In sport, adaptation is plainly domain specific. Athletes may continue to perform while reporting psychological distress (Mifsud et al., 2026; Zhao et al., 2023). Injured athletes may persist in training while returning before recovery is complete (Aldhahi et al., 2026; Madrigal et al., 2016b). High-level athletes may seek support in certain situations but conceal their difficulties in others (Bird and Earl, 2026; Galli and Vealey, 2008; Fletcher and Sarkar, 2012). Therefore, resilience among athletes may not only represent differences in level but also exhibit distinct characteristics across dimensions, contexts, and groups. To understand female athletes’ resilience, it is necessary to examine not only their resilience-related levels and gender differences, but also their associations across psychosocial, performance, and health-related domains.

### Psychological resilience in athletes

Athletes are generally considered to possess high psychological resilience, possibly because competitive sports repeatedly select and train individuals’ ability to perform under pressure (Den Hartigh et al., 2024). Simultaneously, training and competition require athletes to tolerate discomfort and uncertainty (Arnold and Fletcher, 2012), regulate arousal, respond to feedback, and persist after failure (Jones et al., 2002; Walker, 2016). Mental toughness research has typically described these qualities in terms of confidence, control, constancy, persistence, and challenge appraisal (Connaughton et al., 2008; Jones et al., 2002), whereas sport resilience research has emphasized adaptation to stressors through personal and social protective factors (Fletcher and Sarkar, 2012; Sarkar and Fletcher, 2014).

Several studies have found that athletes tend to have higher levels of psychological resilience. For example, Guillen and Laborde (2014) found that athletes from 34 sport disciplines scored higher than non-athletes on a higher-order mental toughness construct, and Nicholls et al. (2009) reported that mental toughness differed by achievement level, age, experience, gender, and sport type. Recently, Hsieh et al. (2024) found a positive association between mental toughness and athletic performance in a meta-analysis. Primary studies have linked mental toughness or resilience-related capacity with basketball (Nemeth et al., 2024; Newland et al., 2013), dance-sport (Jia et al., 2025), and taekwondo performance indicators (Zhao et al., 2023). These findings suggest that athletic training and sport selection may indeed be associated with higher psychological resilience, but this association may also be moderated by factors such as gender and sport type.

However, not all adversity or stress is associated with higher psychological resilience. Dynamic models of resilience propose that adaptation depends on the fit between stress exposure, protective systems, and the domain in which competence is assessed (Denckla et al., 2020; Luthar et al., 2000). Conservation of resources theory reaches a similar conclusion from a stress perspective, demands are most harmful when valued resources are threatened, lost, or insufficiently replenished (Hobfoll, 1989). For example, Fletcher and Sarkar’s (2012) model of Olympic champions did not identify pressure alone as the source of resilience; rather, confidence, motivation, focus, and perceived social support shaped how performers appraised and responded to pressure. Recently, research on child development also challenges the simple claim that adversity builds toughness, because positive childhood experiences have been associated with mental toughness and well-being (Shaw et al., 2023), whereas growth following adversity appears less common than popular narratives imply (Bonner et al., 2025). For female athletes, this distinction matters. High resilience-related scores may reflect adaptive development in supportive sport contexts, whereas low scores may reflect sustained demands without adequate recovery, safety, or support. Associations with outcomes should follow the same logic, resilience is likely to show stronger beneficial associations when athletes have resources that allow them to recover, seek help, and translate coping into performance.

However, previous studies on athletes’ coping mechanisms under stress have not adopted a single concept of resilience. Psychological resilience, mental toughness, and hardiness share a concern with functioning under adversity or pressure, but emphasize partly different aspects of that functioning. Psychological resilience usually refers to adaptation and recovery in the context of adversity or stress (Connor and Davidson, 2003), mental toughness emphasizes confidence, persistence, control, and effective functioning under performance pressure (Gucciardi et al., 2015), and hardiness describes a stress-resistant orientation involving commitment, control, and challenge (Maddi, 2002). Although these concepts related to resilience are not identical, many studies consider them within the framework of psychological resilience. For example, Leppin et al. (2014) included both psychological resilience and hardiness in their meta-analysis of resilience training. In the field of sports psychology, these resilience-related abilities have also been incorporated into the broader model of sports stress adaptation (Fletcher and Sarkar, 2012; Sarkar and Fletcher, 2014). Meanwhile, studies of athletes have shown that while these abilities are related, they are not interchangeable. For example, one study found that in competitive tennis, resilience is positively correlated with psychological toughness; whereas in tests of college athletes, resilience exhibited an imperfect factor structure and item bias (Cowden, 2016; Madrigal et al., 2016a). Therefore, these resilience-related abilities can all be considered within the framework of psychological resilience, while also recognizing their differences.

### Distinctive aspects of psychological resilience in female athletes

The contextual nature of psychological resilience is even more pronounced among female athletes, as athletic demands operate through gendered bodies, roles, and institutional conditions. Differences between female and male athletes in resilience related scores are sometimes interpreted as stable individual differences, but sport and exercise research shows that gender stereotypes can affect participation, perceived competence, and performance evaluation (Chalabaev et al., 2013), and that gender differences in self-confidence have been documented meta-analytically (Lirgg, 1991). In women’s sport, these social processes intersect with body surveillance (Dickens et al., 2023) and disordered eating risk (McManama et al., 2021), relative energy deficiency in sport (Mountjoy et al., 2018), as well as menstrual or endocrine health concerns (Williams et al., 2025), and harassment, mental health needs, and unequal support structures (Mifsud et al., 2026; Reardon et al., 2019).

Mean differences between female and male athletes may therefore reflect several mechanisms, consistent with dynamic and resource-based accounts of resilience (Blanco-Garcia et al., 2021; Denckla et al., 2020; Hobfoll, 1989). They may reflect differences in stress exposure, such as stronger body monitoring in aesthetic or weight-sensitive sports (Chalabaev et al., 2013; Codonhato et al., 2018; Jia et al., 2025; Mountjoy et al., 2018). They may reflect differences in resource access (Hobfoll, 1989), such as coaching support (Karabulut and Akinci, 2025), medical care and financial stability (Reardon et al., 2019), or permission to seek help (Bird and Earl, 2026). They may also reflect measurement content if instruments privilege emotional control, dominance, or pain tolerance (Crust, 2007; Gucciardi et al., 2015; Walker, 2016) over relational coping, self-compassion, or support use (Dickens et al., 2023; Mikesell et al., 2023). Therefore, these situational dynamics may manifest in distinctive ways among female athletes, as their athletic experiences are shaped by gender-based physical, social, and institutional conditions.

For female athletes, women’s sports may be associated with higher resilience-related scores when sport demands are paired with skill development, support, and opportunity. Several studies show this resource pattern directly. For example, Lehmann and Pietsch (2025) reported numerically higher resilience scores among female than male young talented football players. Guillen and Laborde (2014) found only a small female-male difference in resilience among athletes from 34 disciplines, and Leon-Guereno et al. (2020) found similarly small female-male differences among recreational runners. Female-only studies also show how resilience is supported. In adolescent female basketball players, coach-athlete relational quality was positively related to mental toughness (Karabulut and Akinci, 2025). In elite adolescent female netballers, mental toughness buffered the negative effect of controlling coaching on learning (Gucciardi et al., 2017). Among women collegiate athletes during COVID-19, resilience, self-compassion, and social support were associated with lower psychological distress (Mikesell et al., 2023). These findings indicate conditions under which female athletes may show comparable or higher resilience-related capacity, or a stronger protective function of that capacity.

However, opposite patterns may emerge when gendered demands, selection pressures, or limited resources become more prominent. In professional football, Vinu et al. (2025) found that women players reported higher competitive anxiety and lower mental toughness than men. In adolescent dance sport, Jia et al. (2025) found lower mental toughness among girls than boys, and the associations between mental toughness and fitness indicators were consistently slightly weaker among girls. Newland et al. (2013) likewise found that male collegiate basketball players reported higher mental toughness than female players. More importantly, mental toughness predicted performance for male players, whereas starter status was the only significant predictor for female players. Zhao et al. (2023) reported lower resilience scores among female than male Chinese college athletes infected with COVID-19. These studies point to a different interpretation, female athletes may report lower scores, or weaker resilience-outcome links, when the measured capacity is tied to confidence, anxiety, opportunity, or resource loss.

### Potential moderators of resilience in female athletes

Although female athletes share exposure to gendered conditions in sport, resilience-related findings are unlikely to be uniform across athletes, settings, or measures. Age, competitive level, sport type, training context, injury or health adversity may each change what a resilience-related score means (Blanco-Garcia et al., 2021; McManama et al., 2021; Nicholls et al., 2009). These factors may also operate together, because developmental stage, sport demands, institutional resources, and measurement content are not independent in athletes’ lives (Mountjoy et al., 2018; Reardon et al., 2019).

First, moderators may change reported levels and female-male differences. In women practicing mountain sports and climbing, resilience differed by age, with older athletes reporting higher resilient coping than younger athletes, while elite and non-elite women differed in life satisfaction and cognitive anxiety rather than resilience itself (Molero et al., 2025). In judokas, resilience was higher in men than women and was also higher in top-level than non-top-level athletes; the competitive-level difference remained among women after adjustment, although the effect was small (Garrido-Munoz et al., 2024). In an elite female basketball academy, pressure training reduced team-level vulnerabilities and was described by athletes and coaches as improving collective resilience under in-game stressors (Kegelaers et al., 2021). These studies indicate that when assessing the level of psychological resilience in female athletes, factors related to age, competitive level, and training background must also be considered.

Second, moderator variables may alter the relationship between resilience-related abilities and other variables. For example, Lipowski et al. (2016) showed that gender and sport moderated links between resilience and risky behavior; resilience was linked with lower drug-related risk only among athletes, and sport appeared protective for risky sexual behavior in girls but risky in boys. In female college athletes, Wang et al. (2026) found that training stress was negatively related to psychological well-being, with general self-efficacy and psychological resilience carrying a substantial part of the association. In female athletes, Gezer and Korucuk (2026) found that athletic identity was associated with mental toughness both directly and indirectly through sport confidence. Timing may also matter. Williams et al. (2025) found that resilience and well-being were stable across a Division I gymnastics season, but were correlated at the start of competition and postseason, not preseason or midseason. These findings suggest that the role of psychological resilience in female athletes may also vary depending on the sport and individual background.

### The present study

Existing reviews have clarified the conceptual basis of resilience in sport, the performance relevance of mental toughness, and the mental health needs of athletes and young female athletes (Hsieh et al., 2024; McManama et al., 2021; Reardon et al., 2019; Sarkar and Fletcher, 2014). However, these reviews do not resolve two questions central to women’s sport psychology. First, it remains unclear whether female athletes generally report high, comparable, or lower resilience-related capacities than male athletes. Second, it remains unclear whether these capacities are consistently associated with favorable outcomes among female athletes, or whether their associations differ across outcome domains and sport contexts. To answer these questions, research on female athletes has conducted a comprehensive analysis of resilience from dimensions such as psychological resilience, mental toughness, and hardiness. The present meta-analysis therefore estimated female-athlete levels, female-male differences, and female-specific associations with performance, psychological health, coping and adaptation, injury-related outcomes, distress or risk indicators, and social or environmental resources. We further examined whether these estimates varied by age, competitive level, sport context, study characteristics, and construct type, so that the findings could identify not only the average pattern of resilience in female athletes but also the conditions under which resilience-related capacities appear more or less protective.

## Methods

### Literature search

This meta-analysis followed the PRISMA 2020 guidelines (Page et al., 2021) and searched the Web of Science, MEDLINE, ProQuest, PubMed, Scopus, PsycINFO, and IEEE Xplore databases; the final search was completed in July 2026. This review was not prospectively registered. The search comprised two concept blocks: resilience-related constructs (“psychological resilien*” OR “resilien*” OR “sporting resilien*” OR “trait resilien*” OR “hardiness” OR “mental toughness”) and athlete or sport terms (“athlet*” OR “sport performer*” OR “professional sport*” OR “elite sport*” OR “elite athlete*” OR “student athlete*” OR “collegiate athlete*”). For a complete description of the database-specific search strategies, search procedures and PRISMA 2020 Checklist, see the Supplementary materials. In addition, the reference lists of existing meta-analyses and included studies were screened to identify other eligible reports.

### Eligibility and study selection

Studies were eligible if they: (a) reported an empirical quantitative investigation of athletes or sport performers; (b) included female athletes and provided all-female, female-specific, gender-stratified, or female–male comparative data; (c) assessed psychological resilience, sport resilience, mental toughness, or hardiness; and (d) reported sufficient statistics for at least one planned synthesis, female-athlete levels, female-male differences, or female-specific associations with external outcomes. Non-athlete samples, nonpsychological uses of resilience, qualitative or nonempirical publications, and reports without extractable data were excluded. Only English-language full-text articles were included.

**FIGURE 1:**
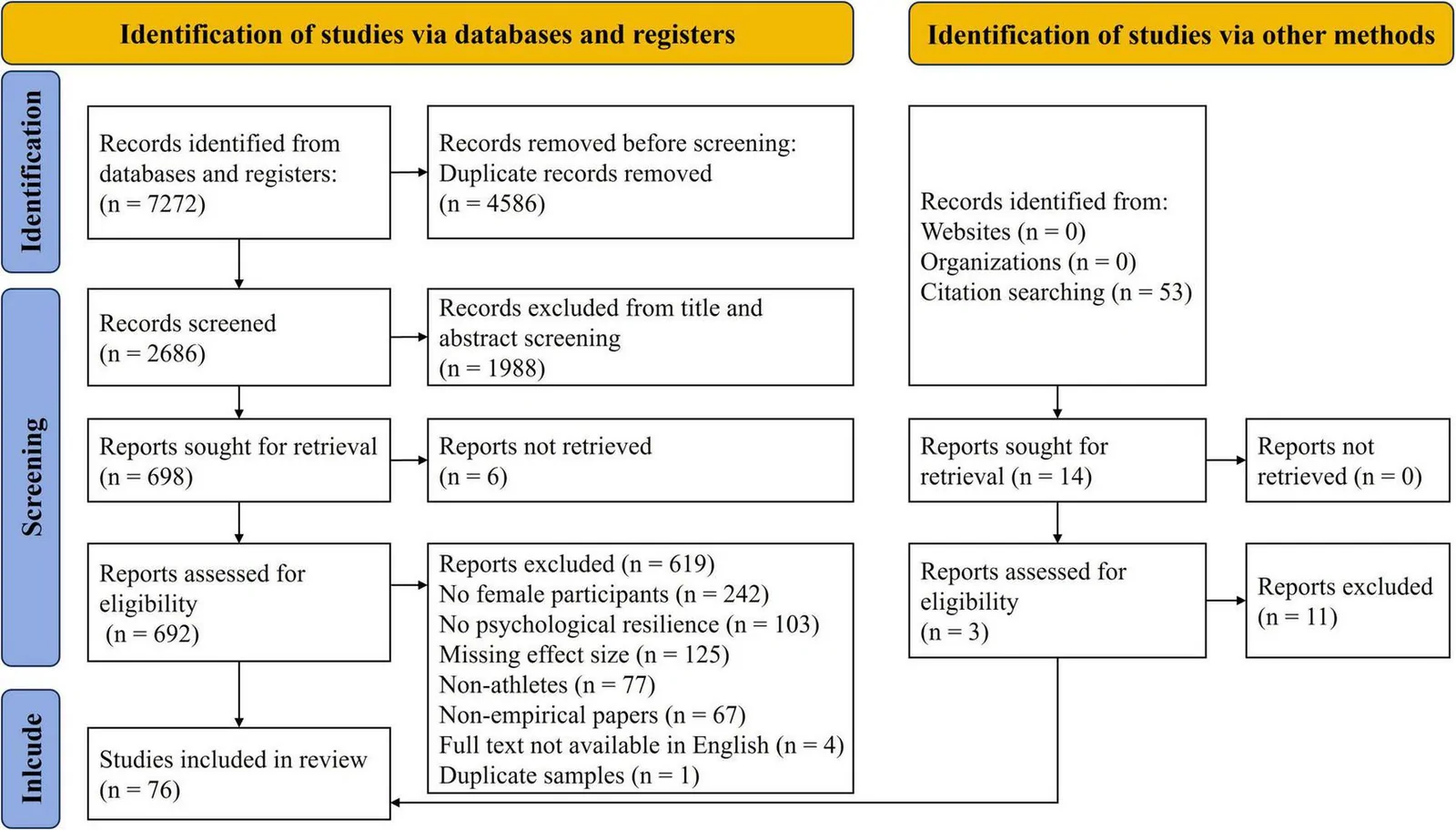
PRISMA flow diagram for study identification, screening, eligibility assessment, and inclusion.

After deduplication, titles and abstracts were screened, followed by full-text assessment against the eligibility criteria. Three psychology students independently conducted the study selection, with all records assessed by two reviewers. Disagreements were resolved through discussion and, when consensus could not be reached, by assessment from a third reviewer. Records were retained for full-text assessment when female-specific data or effect-size eligibility could not be determined from the abstract. The selection process and reasons for full-text exclusion are presented in Figure 1.

### Data extraction and quality assessment

Data were extracted using a structured coding form. Study and sample characteristics included publication year, country or region, design, sample structure, age, sport, competitive level, and relevant injury or adversity context. For each resilience-related measure, the construct, instrument, sample size, mean, standard deviation, possible score range, and female-male comparative statistics were recorded. For association analyses, the resilience measure, outcome, sample size, and correlation or convertible statistic were extracted.

Total-scale scores were prioritized over subscales to provide the broadest representation of each construct and reduce dependence among effects. When a total score was unavailable, eligible subscales were retained and examined in sensitivity analyses. Outcomes were coded into performance or fitness, psychological well-being and resources, coping and adaptation, reduced distress or risk, and social or environmental resources. Effect directions were aligned so that positive associations consistently indicated more adaptive functioning.

Study quality was assessed using a JBI-informed review-specific appraisal tailored to the design of the included quantitative studies. The checklist covered sampling and representativeness, measurement validity, confounder control, missing-data reporting, selective reporting, and the appropriateness of statistical analyses, with design-specific items added for validation, intervention, and longitudinal reports when relevant. Items were coded as yes, partial, no, or not applicable and converted into study-level percentage scores. Across the 76 included reports, quality scores ranged from 50.0 to 81.2, with a mean of 68.7 and a median of 72.2; 41 studies were classified as higher quality and 35 as having some concerns. These quality categories were then used in sensitivity analyses excluding lower-quality studies.

### Effect-size calculation

Three effect-size families were analyzed. First, resilience-related levels were summarized using raw means when studies used the same scoring metric. Following previous research, when instruments differed in score range, study means were converted to percentage-of-maximum-possible (POMP) scores so that heterogeneous measures could be expressed on a common 0–100 metric for pooled descriptive synthesis and cross-study comparison (Cohen et al., 1999). All measures were oriented so that higher scores indicated greater resilience-related capacity. To complement this rescaling, we also computed standardized midpoint-distance (*d*_*mid*_) scores so that pooled means could be interpreted relative to each instrument’s theoretical midpoint. Second, female-male differences were expressed as Hedges’ *g*, calculated as female minus male; negative values therefore indicated lower scores among female athletes.

Third, associations with external outcomes were expressed as correlations. Correlations were transformed to Fisher’s z for analysis and converted back to r for interpretation. To account for the non-independence of multiple correlations reported by the same study, all eligible correlations were first transformed to Fisher’s z. For the overall association synthesis, correlations from the same female sample were averaged on the Fisher’s z scale to form one study-level effect, and the corresponding sampling variances were averaged to obtain the working variance of the aggregated effect. Each study therefore contributed at most one effect to the overall association model. For outcome-domain analyses, correlations were aggregated at the study-by-domain level, multiple correlations from the same study and the same outcome domain were averaged to form one domain-specific effect, whereas effects from different domains were retained for their respective domain analyses. A study could therefore contribute at most one effect to any single domain model but could contribute to more than one domain when distinct eligible outcome domains were reported.

### Statistical analysis

Meta-analyses were conducted in R using the metafor package (Viechtbauer, 2010). Random-effects models were used because the included studies differed in samples, sports, competitive levels, constructs, and outcome domains. The DerSimonian-Laird method-of-moments estimator was retained for the primary analyses to provide a common estimation framework across the three effect-size families. Effect sizes were weighted by the inverse of the sum of within-study variance and τ^2^. For descriptive syntheses of POMP scores, a bounded logit-POMP model was used so that pooled estimates and their uncertainty remained compatible with the 0–100 scale. POMP analyses were re-estimated on the logit-transformed POMP-proportion scale and back-transformed to the 0–100 metric; female-male differences were re-estimated on the Hedges’ *g* scale; and correlation analyses were re-estimated on the Fisher’s *z* scale and back-transformed to Pearson’s *r*. Between-subgroup differences were evaluated using omnibus tests of moderators from mixed-effects meta-regression models. Given the potential for high heterogeneity among studies, sensitivity analyses were conducted for all results. Specifically, for the quality-based analysis, the primary meta-analysis was repeated after excluding studies below the quality threshold and, as a more rigorous validation, after retaining only high-quality studies. Estimator sensitivity analyses re-estimated the models using restricted maximum likelihood with Hartung-Knapp adjustment. Detailed results of the quality sensitivity analysis are available in the public repository https://osf.io/g3hfx/.

Statistical heterogeneity was assessed using Cochran’s Q, I^2^, τ^2^. Prediction intervals were calculated for primary random-effects estimates to characterize the expected range of true effects across comparable sport populations. Given the anticipated and observed heterogeneity, exploratory subgroup analyses were conducted for theoretically and sport-contextually relevant characteristics, including construct type, sport type, age stage, competitive level, injury/adversity context, sample source, and outcome domain where applicable.

Potential publication bias and small-study effects were assessed separately for each primary synthesis using funnel plots, Egger’s regression test, and Begg’s rank correlation test when sufficient studies were available. These analyses were conducted separately because the primary syntheses used different effect-size metrics. Statistical significance was set at *p* < 0.05. Sensitivity analyses evaluated the robustness of the primary findings to study quality, potential sample overlap, and random-effects estimation method. These analyses repeated the main syntheses after excluding lower-quality studies, after removing reports or effects with confirmed or possible sample overlap where relevant, and using REML estimation with Hartung-Knapp adjustment.

## Results

### Publication bias

**FIGURE 2:**
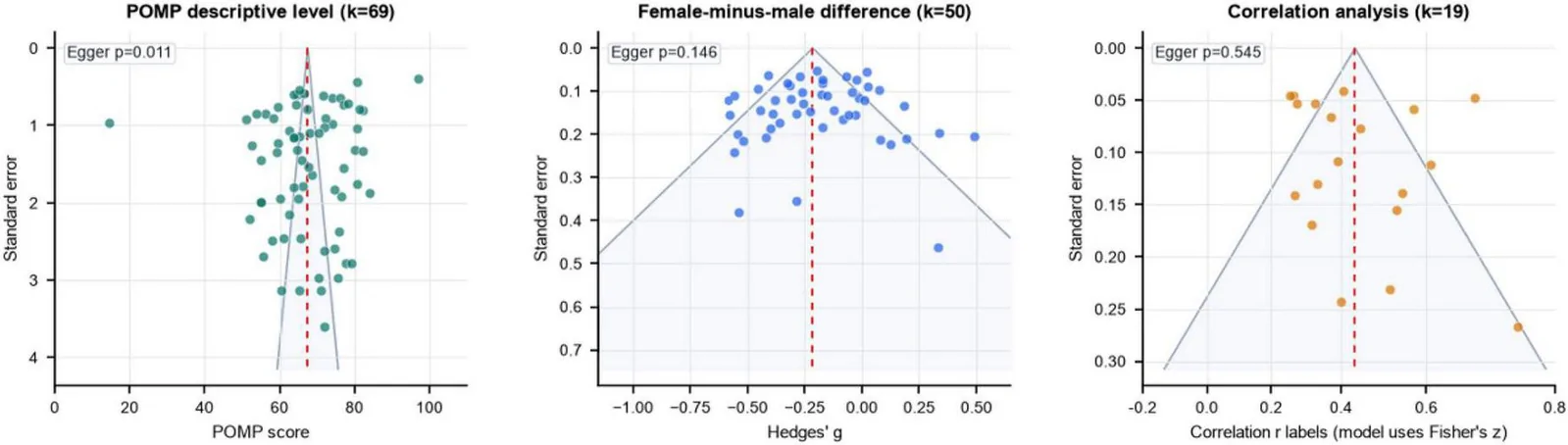
Funnel plots and small-study effect diagnostics for the three primary syntheses.

Funnel plots and small-study effect tests were conducted separately for the three primary syntheses because the effect-size metrics differed across analyses (Figure 2). For the POMP descriptive synthesis, Egger’s regression suggested funnel asymmetry, *b* = −8.14, *p* = 0.011, whereas Begg’s test was not significant, τ = −0.058, *p* = 0.481. Given the very high heterogeneity in this analysis, this pattern was interpreted as possible small-study effects or asymmetry related to between-study differences rather than as definitive evidence of publication bias.

For the female-minus-male synthesis, neither Egger’s test, *b* = −1.05, *p* = 0.146, nor Begg’s test, τ = −0.058, *p* = 0.553, indicated statistically clear funnel asymmetry. A sensitivity analysis excluding one extreme female-minus-male effect produced a similar pooled estimate, *g* = −0.195, 95% CI [−0.255, −0.135], with reduced heterogeneity, *I*^2^ = 69.45%, and no evidence of Egger asymmetry, *p* = 0.744. The main female-male comparison result was therefore not driven by a single outlying study. For the correlation synthesis, Egger’s test, *b* = 0.83, *p* = 0.545, and Begg’s test, τ = 0.216, *p* = 0.196, did not indicate statistically clear funnel asymmetry. Taken together, these checks support the robustness of the female-minus-male and association results, while the descriptive POMP synthesis should be interpreted with greater attention to heterogeneity and possible small-study effects.

### Study characteristics

The final analytic sample comprised 76 studies. Because the available statistics differed across reports, the quantitative syntheses were based on partially overlapping analytic datasets rather than on a single identical set of studies. 68 studies provided female-athlete descriptive data that could be converted to POMP scores. Fifty studies reported data suitable for female-minus-male comparisons. Nineteen studies reported female-only or female-subgroup associations between resilience-related constructs and outcomes; these studies yielded 60 raw correlations, which were reduced to study-level or domain-level effects for the primary analyses. Across the 76 studies with sex-specific sample sizes coded, the sample included at least 14,804 female athletes and 9,719 male athletes.

The studies represented a broad range of sport contexts and resilience-related constructs (Table 1). Mixed or multi-sport samples were most common, followed by team-sport and individual-sport samples. Most studies were conducted in standard sport settings, although a smaller subset examined injury, disability, health-related adversity, COVID-19-related conditions, or other challenging sport contexts. The constructs were coded primarily as psychological resilience or mental toughness, with fewer studies measuring hardiness or closely related resilience-oriented constructs. This evidence structure allowed three linked tests: the absolute level of resilience-related capacities among female athletes, female-male differences, and the associations between resilience-related capacities and female-athlete outcomes.

**TABLE 1:** Characteristics of the included studies and analytic datasets.

References	*N*f	Sports	Type/level/context	Construct/measure	Analyses
Nicholls et al., 2009	NR	Multiple sports	Mixed	MT (MTQ48)	D, FM
Crust and Keegan, 2010	36	Multiple sports	Mixed; Elite/pro	MT (MTQ48)	D, FM
Hosseini and Besharat, 2010	43	Multiple sports	Mixed; Collegiate	Resilience (CD-RISC)	D, FM
Newland et al., 2013	105	Basketball	Team; Collegiate	MT proxy; MT (PPI-A)	D, FM
Cardoso and Sacomori, 2014	58	Multiple sports	Mixed; Elite/pro; injury/health	Resilience (RS)	D, FM
Guillen and Laborde, 2014	486	Multiple sports	Mixed	Resilience (RS-15)	D, FM
Bingol and Bayansalduz, 2016	313	Multiple sports	Mixed; NR	Resilience (ERS)	D, FM
Cowden, 2016	174	Tennis	Indiv.; Competitive	MT (SMTQ)	D, FM
Lipowski et al., 2016	278	Multiple sports	Mixed; Youth/dev	Resilience (SPP-18)	R
Madrigal et al., 2016a	322	Multiple sports	Mixed; Collegiate	Hardiness (DRS)	D, FM
Madrigal et al., 2016b	190	Rugby	Mixed; Collegiate	MT	D, R
Walker, 2016	484	Field hockey	Team; Youth/dev	MT (SMTQ)	D, R
Gucciardi et al., 2017	232	Netball	Team; Elite/pro	MT (MTI)	D, R
Wilson and Madrigal, 2017	54	Multiple sports	Mixed; Elite/pro; injury/health	MT	R
Codonhato et al., 2018	8	Gymnastics	Indiv./team; Elite/pro; injury/health	Resilience (CD-RISC-10)	D
Chacón-Cuberos et al., 2019	320	Physical education/activity	Mixed/NR; NR/mixed	Resilience (CD-RISC)	D, FM
Eryücel, 2019	24	Wrestling	Indiv.; NR	Resilience (RSA)	D, FM
Goddard et al., 2019	9	Running/endurance	Indiv.; Ultra-endurance	MT (SMTQ)	D, FM
Gupta and Sudhesh, 2019	16	Football	Team; Collegiate; injury/health	Resilience (BU-RS)	D, FM
Kristjansdottir et al., 2019	142	Football/soccer	Team; Elite/pro	MT (SMTQ)	D
Walker, 2019	486	Field hockey	Team; Youth/dev	MT (SMTQ)	R
Karademir and Gençay, 2020	104	Multiple sports	Mixed; Youth/dev	Resilience (PRS)	D, FM
Küçük Kiliç, 2020	54	Karate	Indiv.; NR	Resilience (BRS)	D, FM
Leon-Guereno et al., 2020	465	Running	Indiv.; Amateur/club; injury/health	Resilience (CD-RISC-10)	D, FM
Onturk et al., 2020	NR	Multiple sports	Mixed; Collegiate	Resilience (BRS)	D, FM
Yasar and Turgut, 2020	63	Judo	Indiv.; Elite/pro	MT	D, FM
Blanco-Garcia et al., 2021	458	Multiple sports	Mixed	Resilience (BRS)	D, FM
Cano-Noguera et al., 2021	50	Futsal and lifeguard	Mixed; Youth/dev	Resilience (CD-RISC-10)	D, FM
Cowden et al., 2021	99	Tennis	Indiv.; Youth/dev	MT (MTInv)	D, FM
Kegelaers et al., 2021	18	Basketball	Team; Elite/pro	Resilience (CD-RISC-10)	D
Lorenzo Calvo et al., 2021	47	Basketball	Team; NR	Resilience (BRS)	D, FM
Martin et al., 2021	3924	Multiple sports	Mixed; Collegiate	Resilience (BRS)	D, FM
Zarić et al., 2021	38	Basketball	Team; Elite/pro	MT (MTQ48)	D, R
Ajilchi et al., 2022	45	Multiple sports	Mixed; Collegiate	MT (SMTQ)	D
Doğan et al., 2022	201	Multiple sports	Mixed; Competitive	MT (SMTQ)	D, FM
López-Mora et al., 2022	34	Multiple sports	Mixed; NR	MT (Spanish)	D, FM
Tian et al., 2022	692	Multiple sports	Mixed; Youth/dev	MT	D, FM
Cooley A. P. et al., 2023	28	Lacrosse	Team; Collegiate	Hardiness (DRS-15)	D
Cooley A. et al., 2023	17	Lacrosse	Team; Elite/pro; injury/health	Hardiness (DRS-15)	D
Dickens et al., 2023	354	17 NCAA sports	Mixed; Collegiate	Resilience (BRS)	D, R
Patenteu et al., 2023	22	Martial Arts	Indiv.; Amateur/club; injury/health	Resilience	D, FM
Wang, 2023	105	Tennis	Indiv.; Elite/pro	Resilience (PRS)	D, FM
Yazıcı et al., 2023	138	Multiple sports	Mixed; Collegiate	Resilience (BRS)	D, FM
Zhao et al., 2023	53	Football	Team; Collegiate; injury/health	Resilience	FM
Garrido-Munoz et al., 2024	233	Judo	Indiv.; Mixed	Resilience (CD-RISC-10)	D, FM
Griffith et al., 2024	54	Hockey/lacrosse	Team; Elite/pro	MT (MTI)	D, R
Nemeth et al., 2024	20	Basketball	Team; Elite/pro	MT (SMTQ)	D, R
Rubén and Joaquín, 2024	NR	Basketball	Team; Elite/pro	MT (CNPD/APNQ)	D, FM
Sehrawat et al., 2024	155	Multiple sports	Mixed; mixed context	Resilience (CD-RISC)	D, FM
Wandik et al., 2024	36	Multiple sports	Mixed; Elite/pro	MT (MTInv)	D, FM
Xiang et al., 2024	212	Multiple sports	Mixed; Level I+	MT	D, FM
Yarayan et al., 2024	139	Running	Indiv.; Elite/pro	MT	D, FM
Zhao et al., 2024	55	Taekwondo	Indiv.; Elite/pro	Resilience (CD-RISC-10)	R
Di Corrado et al., 2025	43	Taekwondo	Indiv.; Elite/pro	Resilience (NR)	D, FM
Jia et al., 2025	174	Dance	Indiv./pair; Youth/dev	MT (SMTQ)	D, FM, R
Karabulut and Akinci, 2025	366	Basketball	Team; Youth/dev	MT (SMTQ)	D, R
Lehmann and Pietsch, 2025	59	Football/soccer	Team; Youth/dev	Resilience (RS-11)	D, FM
Lorenco-Lima et al., 2025	89	Brazilian jiu-jitsu	Indiv.; Mixed	Resilience (BRS)	D, FM, R
Molero et al., 2025	91	Mountain sports and climbing	Mixed; Elite/pro	Resilience (NR)	D
Papadakis et al., 2025	NR	Multiple sports	Mixed; Collegiate	MT (MTI)	D, FM
Stoyanova et al., 2025	NR	Multiple sports	Mixed; Elite/pro	Resilience (BRS)	D, FM
Sundgot-Borgen et al., 2025	NR	Multiple sports	Mixed; Elite/pro	Resilience (READ)	D, FM
Tawil et al., 2025	69	Multiple sports	Mixed; Elite/pro; injury/health	MT (SMTQ)	D, FM
Vinu et al., 2025	32	Football	Team; Elite/pro	MT	FM
Williams et al., 2025	17	Gymnastics	Indiv.; Elite/pro	Resilience (BRS)	R
Workman and Stamatis, 2025	15	Field hockey	Team; Elite/pro; injury/health	MT (MTI)	D
Zhang and Ma, 2025	NR	Multiple sports	Mixed; Youth/dev	MT (SMTQ)	R
Zhou and Stefan, 2025	84	Dance	Indiv.; Elite/pro; excl. disorder	MT (SMTQ)	D, FM, R
Aldhahi et al., 2026	60	Multiple sports	Mixed; Collegiate; injury/health	Resilience (BRS)	D
Bird and Earl, 2026	146	Multiple sports	Mixed; NR	Resilience (CD-RISC-10)	D, FM
Chen et al., 2026	171	Multiple sports	Mixed; Collegiate	Resilience (CD-RISC)	D, FM
Gezer and Korucuk, 2026	449	Multiple sports	Mixed; Elite/pro	MT (SMTQ)	D, R
Girginer et al., 2026	9	Swimming	Indiv.; Youth/dev	MT (SMTQ)	D, FM
Mifsud et al., 2026	240	Football	Team; Elite/pro	Resilience (CD-RISC)	D, FM
Rowan et al., 2026	393	Multiple sports	Mixed; mixed context	Resilience (CD-RISC-10; BRS)	D
Wang et al., 2026	604	Multiple sports	Mixed; Collegiate	Resilience (PRS)	D, R

D, descriptive/POMP synthesis; FM, female-minus-male comparison synthesis; R, female-athlete correlation synthesis; Nf, number of female athletes; MT, mental toughness; NR, not reported. Type/level/context reports sport type and competitive level; non-standard contexts are noted where coded.

### Levels of resilience-related capacities among female athletes

To examine the level of resilience-related capacities among female athletes, descriptive statistics were synthesized for the mean scores of eligible resilience-related measures. In the primary one-effect-per-study model, the pooled POMP estimate was 68.53, 95% CI [66.13, 70.83], based on 68 studies and 11,978 female athletes (Table 2). The corresponding *d*_*mid*_ was 1.45, 95% CI [1.25, 1.65], indicating that female athletes’ resilience-related scores lay above the theoretical midpoint of the scales used.

**TABLE 2:** Random-effects POMP estimates for resilience-related capacities in female athletes.

Analysis	Group	*k*	*N*	POMP [95% CI]	*d*_mid_	*I*^2^ (%)	PI
Overall	Overall	68	11978	68.53 [66.13, 70.83]	1.45	98.75	47.21, 84.13
Construct	Psychological resilience	35	6596	69.20 [65.83, 72.38]	1.39	98.74	47.53, 84.79
	Mental toughness	30	4813	66.31 [62.87, 69.58]	1.25	98.60	46.42, 81.72
	Hardiness	3	569	81.03 [43.92, 95.88]	4.75	99.51	12.63, 99.21
Sample source	All-female	22	4465	67.87 [65.33, 70.32]	1.49	96.82	55.51, 78.15
	Mixed-sex	46	7513	68.78 [65.30, 72.06]	1.39	99.03	43.13, 86.48
Sport type	Team sports	17	1941	69.65 [66.47, 72.65]	1.91	96.16	55.79, 80.67
	Individual sports	17	1986	69.66 [64.80, 74.12]	1.39	97.91	47.90, 85.15
	Mixed/multi-sport	34	8051	67.40 [63.58, 71.00]	1.24	99.19	43.45, 84.76
Age stage	Adult/college-age	39	6704	68.72 [65.57, 71.71]	1.50	98.66	47.49, 84.22
	Minor/adolescent	18	3223	68.01 [62.08, 73.40]	1.43	99.07	40.85, 86.74
Competition level	Elite/professional	24	2544	70.89 [67.47, 74.09]	1.61	96.98	52.75, 84.16
	Collegiate/university	15	2762	67.78 [62.55, 72.60]	1.40	98.58	45.85, 83.94
	Youth/developmental	9	2037	70.57 [65.87, 74.86]	1.54	97.87	55.10, 82.41
Injury/adversity context	Standard sport context	48	9201	68.63 [65.60, 71.50]	1.46	99.00	45.91, 84.93
	Injury/health adversity	9	736	68.72 [63.18, 73.77]	1.58	93.80	51.00, 82.25

POMP, percentage of maximum possible; PI, prediction interval. Estimates are based on one effect per study; mixed, not reported, and other categories were retained when the available study information did not support assignment to a single substantive category. Complete results for all subgroup analyses are provided in Supplementary Appendix 2.

Subgroup estimates by construct were close in magnitude for psychological resilience and mental toughness. Studies measuring psychological resilience produced a pooled POMP score of 69.20, 95% CI [65.83, 72.38], *d*_*mid*_ = 1.39, *k* = 35, whereas studies measuring mental toughness produced a pooled estimate of 66.31, 95% CI [62.87, 69.58], *d*_*mid*_ = 1.25, *k* = 30. Hardiness showed a numerically higher estimate, 81.03, but the confidence interval was very wide, 95% CI [43.92, 95.88], *d*_*mid*_ = 4.75, *k* = 3, and this subgroup was too small for a stable inference. Thus, the descriptive conclusion that female athletes reported above-midpoint resilience-related scores was not driven by a single construct family. Psychological resilience and mental toughness showed broadly similar POMP estimates, whereas the hardiness estimate should be interpreted cautiously because it was based on only three studies and had wide uncertainty.

Sample source did not meaningfully change the descriptive pattern of female athletes’ resilience-related levels. All-female samples showed a pooled POMP score of 67.87, 95% CI [65.33, 70.32], *d*_*mid*_ = 1.49, *k* = 22, and female subgroups extracted from mixed-sex studies showed a similar estimate of 68.78, 95% CI [65.30, 72.06], *d*_*mid*_ = 1.43, *k* = 46. This similarity indicates that the descriptive conclusion was not driven only by studies designed around female athletes. At the same time, heterogeneity remained high within both subgroups, especially among mixed-sex studies, indicating that the reporting source did not account for the broad variation in resilience-related scores.

Sport type, age stage, competitive level, and adversity context showed more descriptive variation, but most subgroup confidence intervals overlapped. Team-sport samples had a pooled POMP score of 69.65, 95% CI [66.47, 72.65], *d*_*mid*_ = 1.91, *k* = 17; individual-sport samples showed 69.66, 95% CI [64.80, 74.12], *d*_*mid*_ = 1.39, *k* = 17; and mixed or multi-sport samples showed 67.40, 95% CI [63.58, 71.00], *d*_*mid*_ = 1.24, *k* = 34. Adult or college-age samples and minor or adolescent samples were almost identical, with pooled estimates of 68.72 and 68.01, respectively. Competition level showed the clearest numerical pattern, elite or professional samples, 70.89, 95% CI [67.47, 74.09], *d*_*mid*_ = 1.61, *k* = 24, and youth or developmental samples, 70.57, 95% CI [65.87, 74.86], *d*_*mid*_ = 1.54, *k* = 9, were higher than collegiate or university samples, 67.78, 95% CI [62.55, 72.60], *d*_*mid*_ = 1.40, *k* = 15. Studies conducted in injury or health-adversity contexts did not show lower scores than standard sport-context studies, 68.72 versus 68.63. These subgroup results are best interpreted as descriptive patterns because heterogeneity remained large within most categories.

### Gender differences in athletes’ resilience-related capacities

To further explore the levels of psychological resilience among female athletes, we conducted a comparison test on all studies that included samples comparing men and women, in order to examine the relative levels of female athletes compared to male athletes. The female-male comparison synthesis included 50 studies with 7,677 female athletes and 10,939 male athletes. Effect sizes were coded as female minus male, so negative values indicate lower resilience-related scores among female athletes. The pooled random-effects estimate showed a small but statistically reliable female-minus-male difference, Hedges’ *g* = −0.22, 95% CI [−0.29, −0.14] (Table 3). This finding indicates that female athletes reported slightly lower resilience-related scores than male athletes in the available comparative literature. The prediction interval, however, ranged from −0.67 to 0.24, and heterogeneity was substantial, *I*^2^ = 80.91%, τ^2^ = 0.051. The average difference therefore favored male athletes, but the direction and size of the difference were not expected to be the same in every sport population.

**TABLE 3:** Gender differences in in resilience-related capacities.

Analysis	Group	*k*	*N* female	*N* male	*g* [95% CI]	*I*^2^ (%)	QM
Overall	Overall	50	7677	10939	−0.22 [−0.29, −0.14]	80.91	
Construct	Mental toughness	20	2635	3284	−0.33 [−0.49, −0.16]	87.84	2.05
	Resilience	29	4720	7453	−0.18 [−0.26, −0.11]	69.43	
Sport type	Individual	16	1781	3329	−0.21 [−0.34, −0.08]	72.06	5.63^†^
	Mixed/multi-sport	26	5270	6642	−0.17 [−0.24, −0.10]	69.35	
	Team	8	626	968	−0.73 [−1.20, −0.26]	93.62	
Age stage	Adult/college-age	28	4418	6441	−0.32 [−0.43, −0.21]	84.72	5.35*
	Minor/adolescent	13	2085	2519	−0.11 [−0.24, 0.02]	71.02	
	Mixed/not reported	9	1174	1979	−0.08 [−0.23, 0.07]	72.56	
Competition level	Collegiate/university	10	1332	1687	−0.28 [−0.42, −0.15]	62.72	1.71
	Elite/professional	14	1498	1894	−0.37 [−0.61, −0.13]	89.87	
	Youth/developmental	7	1187	1513	−0.06 [−0.28, 0.16]	79.71	
Injury/adversity context	Injury/health adversity	6	683	1854	−0.12 [−0.28, 0.04]	44.04	0.13
	Standard sport context	36	6020	8115	−0.22 [−0.31, −0.13]	84.33	

Hedges’ *g* was calculated as female minus male; negative values indicate lower scores among female athletes. QM values are omnibus between-subgroup moderator tests. Complete results for all female-minus-male subgroup analyses are provided in Supplementary Appendix 2.

† *p* < 0.1; *p < 0.05.

Construct-specific estimates suggested a numerically larger female-minus-male difference for mental toughness, *g* = −0.33, 95% CI [−0.49, −0.16], *k* = 20, than for psychological resilience, *g* = −0.18, 95% CI [−0.26, −0.11], *k* = 29. However, the formal between-subgroup test was not statistically significant, QM(1) = 2.05, *p* = 0.152. Therefore, construct type did not provide clear statistical evidence of moderation. The more negative estimate for mental toughness should be interpreted as a descriptive pattern rather than as evidence that female-male differences are reliably larger for mental toughness than for psychological resilience.

Sport type showed a stronger numerical contrast, but the omnibus moderator test did not reach the conventional threshold for statistical significance, QM(2) = 5.63, *p* = 0.060. Mixed or multi-sport samples showed a small female-minus-male difference, *g* = −0.17, 95% CI [−0.24, −0.10], *k* = 26, and individual-sport samples showed a similar estimate, *g* = −0.21, 95% CI [−0.34, −0.08], *k* = 16. Team-sport samples showed a larger negative estimate, *g* = −0.73, 95% CI [−1.20, −0.26], *k* = 8, but this subgroup also had very high heterogeneity, *I*^2^ = 93.62%, and a wide prediction interval, −2.04 to 0.59. Accordingly, the team-sport result is best treated as an exploratory pattern that requires further testing in larger and more homogeneous samples.

Age stage was the only subgroup variable that showed statistically supported moderation. Adult or college-age samples showed a larger female-minus-male difference, *g* = −0.32, 95% CI [−0.43, −0.21], *k* = 28, than minor or adolescent samples, *g* = −0.11, 95% CI [−0.24, 0.02], *k* = 13, QM(1) = 5.35, *p* = 0.021. Mixed or not-reported age samples showed a small estimate, *g* = −0.08, 95% CI [−0.23, 0.07], *k* = 9, and are reported descriptively. This result indicates that the female-minus-male difference was more evident in adult or college-age samples than in younger samples.

By contrast, competitive level and adversity context did not show statistically supported between-subgroup differences. Elite or professional samples, *g* = −0.37, 95% CI [−0.61, −0.13], *k* = 14, and collegiate or university samples, *g* = −0.28, 95% CI [−0.42, −0.15], *k* = 10, showed negative estimates, whereas youth or developmental samples showed a weaker estimate, *g* = −0.06, 95% CI [−0.28, 0.16], *k* = 7. However, the moderator test for competitive level was not significant, QM(2) = 1.71, *p* = 0.426. Similarly, standard sport-context studies showed a pooled estimate of *g* = −0.22, 95% CI [−0.31, −0.13], *k* = 36, and injury or health-adversity studies showed a smaller and statistically unclear estimate, *g* = −0.12, 95% CI [−0.28, 0.04], *k* = 6, but the between-subgroup test was not significant, QM(1) = 0.13, *p* = 0.717. These findings indicate that age stage, rather than construct type, competitive level, sport type, or adversity context, provided the clearest statistical evidence of variation in female-male differences.

### Associations across psychosocial, performance, and health domains

To examine how resilience-related capacities were associated with female athletes’ functioning, we synthesized correlations across psychosocial, performance, and health-related domains. Nineteen studies contributed 60 raw correlations based on all-female samples or female subgroups from mixed-sex studies. After aligning effect directions so that positive values indicated more adaptive outcomes, correlations from the same study and sample were averaged on the Fisher’s z scale to form one study-level effect for the overall synthesis. For domain-specific analyses, correlations were instead aggregated within each study and outcome domain, allowing a study to contribute at most one effect to any given domain. The overall analysis therefore comprised 19 study-level effects and yielded a moderate positive association, *r* = 0.44, 95% (Table 4). The prediction interval remained positive, 0.08–0.69, but heterogeneity was substantial, *I*^2^ = 87.52%, τ^2^ = 0.036. Thus, higher resilience-related scores were generally associated with more adaptive indicators among female athletes, although the magnitude of the association varied across domains.

**TABLE 4:** Random-effects correlations between resilience and psychosocial, performance, and health-related domains.

Analysis	Group	*k*	*N* range	*r* [95% CI]	*I*^2^ (%)	PI
Overall	Overall	19	15-604	0.44 [0.35, 0.51]	87.52	0.08, 0.69
Broad domain	Coping/adaptation	3	15-89	0.42 [0.25, 0.57]	21.63	0.20, 0.61
	Performance/fitness	6	20-174	0.46 [0.35, 0.56]	36.22	0.26, 0.62
	Well-being/resources	9	17-604	0.46 [0.32, 0.58]	94.94	−0.01, 0.76
	Reduced distress/risk	6	62-604	0.37 [0.23, 0.49]	89.16	0.02, 0.64
	Social resources	3	232-366	0.29 [0.03, 0.51]	94.19	−0.22, 0.68
Specific domain	Coping/adaptation	2	15-47	0.50 [0.29, 0.67]	0	0.29, 0.67
	Physical fitness	2	84-174	0.55 [0.32, 0.72]	78.31	0.16, 0.79
	Positive mental health	9	17-604	0.47 [0.33, 0.59]	94.74	0.02, 0.76
	Reduced mental symptoms	4	89-486	0.39 [0.18, 0.56]	93.09	−0.08, 0.71
	Sport performance	3	20-55	0.45 [0.29, 0.59]	0	0.29, 0.59
Construct	Mental toughness	13	15-486	0.44 [0.33, 0.54]	90.64	0.02, 0.73
	Resilience	6	17-604	0.40 [0.30, 0.49]	60.40	0.19, 0.58

Positive *r*-values indicate more adaptive outcomes after direction adjustment. Multiple raw correlations were aggregated to study-level or domain-level effects for the primary models.

Broad outcome domains showed positive associations in each category examined. The pooled correlations were similar for psychological well-being and resources, *r* = 0.46, 95% CI [0.32, 0.58], *k* = 9; performance or fitness outcomes, *r* = 0.46, 95% CI [0.35, 0.56], *k* = 6; and coping or adaptation outcomes, *r* = 0.42, 95% CI [0.25, 0.57], *k* = 3. Associations were somewhat smaller for reduced distress or risk outcomes, *r* = 0.37, 95% CI [0.23, 0.49], *k* = 6, and social or environmental resources, *r* = 0.29, 95% CI [0.03, 0.51], *k* = 3. These estimates indicate that resilience-related capacities were not associated only with psychological well-being. They were also related to performance, fitness, coping, and lower maladaptive outcomes in female-athlete samples.

**FIGURE 3:**
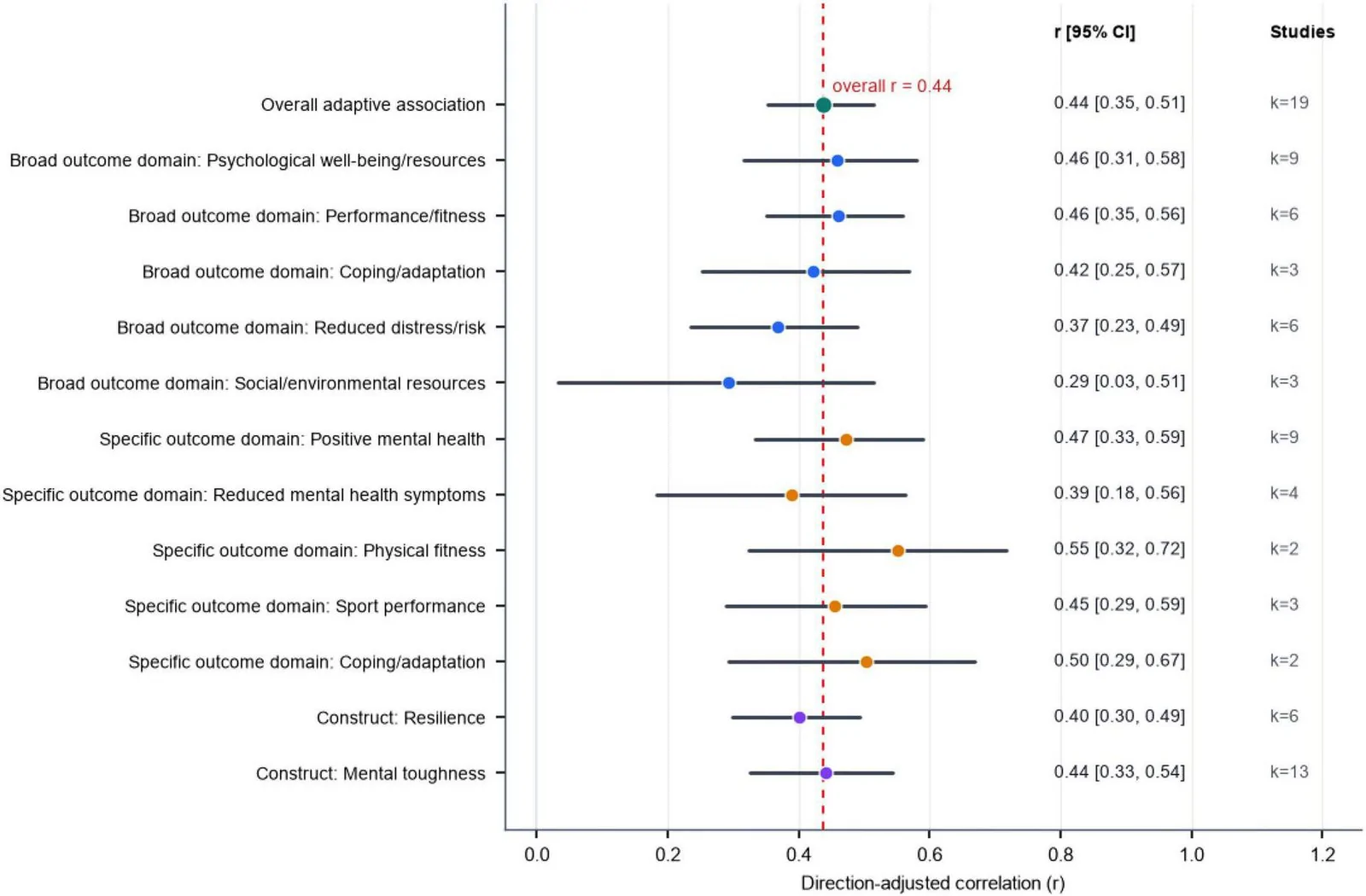
Random-effects forest plot for correlations between resilience and psychosocial, performance, and health-related domains.

The specific-domain analysis showed a similar pattern while also revealing differences in precision (Figure 3). Positive mental health had the largest evidence base, *r* = 0.47, 95% CI [0.33, 0.59], *k* = 9. Reduced mental health symptoms showed a positive direction-adjusted association, *r* = 0.39, 95% CI [0.18, 0.56], *k* = 4. Sport performance, *r* = 0.45, 95% CI [0.29, 0.59], *k* = 3, and coping or adaptation, *r* = 0.50, 95% CI [0.29, 0.67], *k* = 2, showed moderate positive estimates with low observed heterogeneity, although the number of studies was small. Physical fitness showed the largest point estimate, *r* = 0.55, 95% CI [0.32, 0.72], *k* = 2, but this estimate should be interpreted cautiously because it was based on only two studies. The broad pattern was therefore consistent across domains, but the strength of evidence was uneven.

Associations were also similar across the two main construct families. Mental toughness showed a pooled association of *r* = 0.44, 95% CI [0.33, 0.54], *k* = 13, and psychological resilience showed *r* = 0.40, 95% CI [0.30, 0.49], *k* = 6. The mental toughness subgroup showed greater heterogeneity, *I*^2^ = 90.64%, whereas the resilience subgroup showed lower, but still meaningful, heterogeneity, *I*^2^ = 60.40%. These findings indicate that both construct families were positively associated with adaptive indicators among female athletes. They also show why outcome classification is important. The overall association was positive, but the included outcomes represented different domains, including performance or fitness, psychological well-being and resources, coping and adaptation, reduced distress or risk, and social or environmental resources. The estimates therefore should be interpreted as domain-specific associations rather than as a single global effect.

### Sensitivity analyses

Two complementary sensitivity-analysis families were used to evaluate the robustness of the primary findings. First, in the sensitivity analysis of study quality, all included studies scored at least 50% in the quality assessment. In higher-quality studies only, the pooled POMP estimate was 69.12, 95% CI [65.62, 72.41] (*k* = 39), and the female-minus-male synthesis remained negative, Hedges’ *g* = −0.20, 95% CI [−0.28, −0.12] (*k* = 37). A higher-quality-only correlation estimate was not estimable because none of the 19 studies contributing to the association synthesis met the higher-quality threshold.

Second, in the sensitivity analysis of the estimator using the restricted maximum likelihood method with Hartung-Knapp adjustment. REML-Hartung-Knapp analyses produced results comparable to the primary DerSimonian-Laird models. The REML-Hartung-Knapp estimate was 68.58, 95% CI [65.30, 71.69] for the POMP synthesis, *g* = −0.22, 95% CI [−0.34, −0.09] for the female-minus-male synthesis, and *r* = 0.44, 95% CI [0.35, 0.51] for the association synthesis. Thus, the direction and substantive interpretation of the three primary findings were stable across the estimator sensitivity analyses.

Thus, the direction and substantive interpretation of the three primary findings were stable across the estimator sensitivity analyses. Complete results for the sensitivity analyses are provided in https://osf.io/g3hfx/.

## Discussion

This meta-analysis comprehensively quantifies resilience-related abilities in female athletes for the first time by examining their resilience-related levels, gender differences, and associations across psychosocial, performance, and health-related domains. Combining 76 studies, this meta-analysis found that female athletes’ psychological resilience is not simply a matter of being more vulnerable or more resilient. Female athletes’ absolute scores on resilience-related measures were generally located in the upper half of the available scale ranges and above the theoretical midpoints of the measures used. At the same time, direct female-minus-male comparisons showed a small but reliable disadvantage for female athletes, with age stage providing the clearest statistically supported source of variation. Other subgroup patterns, including construct type, competitive level, and sport type, were informative but should be interpreted descriptively because formal between-subgroup tests did not confirm reliable moderation. The correlational synthesis added a second layer to the interpretation. Resilience-related capacities were moderately associated with adaptive indicators among female athletes, with positive associations observed across performance or fitness, psychological well-being and resources, coping and adaptation, reduced distress or risk, and social or environmental resources. Taken together, the results indicate that female athletes often report resilience-related scores above the theoretical midpoints of the measures used, but that this capacity is shaped by gendered sport conditions and is functionally meaningful for both performance and health.

### Resilience levels in female athletes

The current female-athlete evidence base is concentrated mainly in psychological resilience and mental toughness, whereas hardiness is represented by relatively few studies. The descriptive findings are consistent with the idea that competitive sport can be associated with, or select for, resilience-related capacities. Athletes repeatedly train under evaluation, fatigue, feedback, uncertainty, and selection pressure (Fletcher and Sarkar, 2012; Jones et al., 2002), and sport-specific theories of mental toughness and resilience describe these environments as contexts in which confidence, emotional regulation, challenge appraisal, attentional control, persistence, and social support are practiced over time (Connaughton et al., 2008; Sarkar and Fletcher, 2014). Prior empirical work supports this expectation. Guillen and Laborde (2014) found higher mental toughness in athletes than in non-athletes across 34 sport disciplines, and Nicholls et al. (2009) found that mental toughness varied by achievement level, age, experience, gender, and sport type. The present finding extends this literature by showing that female athletes, considered on their own rather than as a subgroup within general athlete samples, report resilience-related levels that are above the theoretical midpoint of the measures used in the field.

Nevertheless, this does not mean that female athletes have a general psychological advantage. The descriptive synthesis showed very high heterogeneity and a wide prediction interval, indicating that a female athlete drawn from one sport, developmental stage, country, or measurement tradition may not resemble a female athlete drawn from another. This variability fits dynamic models of resilience, which define resilience as adaptation within specific risk and resource systems rather than as a trait with the same meaning across contexts (Denckla et al., 2020; Luthar et al., 2000; Masten, 2001). It also fits conservation of resources theory, repeated demands may support adaptation when athletes have recovery, autonomy, safety, and social support, but the same demands may become harmful when resources are lost or unavailable (Hobfoll, 1989). In other words, sport participation may help explain why the average level is not low, whereas sport-specific and gendered resource conditions help explain why the scores vary so widely.

The current findings not only align with previous meta-analyses but also expand our understanding of psychological resilience. Hsieh et al. (2024) showed that mental toughness is associated with athletic performance; Lin et al. (2017) summarized links between mental toughness, performance, well-being, and personality across domains, and Hu et al. (2015) found that trait resilience is related to better mental health. Those reviews established the functional relevance of resilience-related constructs, but they did not determine whether female athletes themselves show relatively high or low levels. The present study further showed that female athletes’ resilience-related scores were generally above the theoretical midpoint of the measures used, although this pattern depended on sport exposure, measurement content, developmental stage, and resource availability.

### Gender differences in athletes’ resilience-related capacities

The female-minus-male synthesis showed that female athletes scored slightly lower than male athletes on resilience-related measures. This finding does not contradict the descriptive result. Thus, female athletes may appear resilient in absolute terms while still showing a modest average disadvantage in direct female-minus-male comparisons. This pattern is consistent with the broader sport psychology literature showing that gender stereotypes shape sport participation, competence beliefs, and performance evaluation (Chalabaev et al., 2013), while gender differences in self-confidence have also been documented across physical activity contexts (Lirgg, 1991).

Therefore, compared to male athletes, female athletes’ lower psychological resilience may be a situational indicator rather than evidence of an inherent deficiency in women. Women’s sport includes stressors that are unevenly distributed across athletes, including body surveillance and disordered eating risk (McManama et al., 2021), RED-S and menstrual health concerns (Mountjoy et al., 2018), and harassment risk and unequal material or psychosocial support (Reardon et al., 2019). These conditions can reduce resilience-related functioning by increasing demands and reducing resources. They can also affect how athletes interpret and report toughness. In some settings, the behaviors rewarded as toughness may include hiding distress, tolerating pain, or avoiding help-seeking. The present female-male comparison result is therefore more consistent with a gendered sport ecology account than with an individual-deficit account.

The construct subgroup findings suggest one possible source of inconsistency, although they do not establish construct type as a statistically confirmed moderator. The female-minus-male estimate was numerically more negative for mental toughness than for psychological resilience, but the formal moderator test was not significant. This distinction matters because mental toughness measures often emphasize confidence, control, constancy, persistence, and pressure tolerance (Crust, 2007; Gucciardi et al., 2015), whereas resilience measures more often emphasize recovery, adaptation, or the capacity to bounce back after stress (Smith et al., 2008). Thus, construct choice may still influence how gendered sport adaptation is represented, especially when measures emphasize traits that are culturally tied to confidence, emotional control, and pressure execution. The present findings therefore support treating construct type as an important descriptive and methodological feature, rather than as a confirmed source of female-male difference.

Age stage provides the clearest evidence that female-male differences in resilience-related scores are not developmentally fixed. This interpretation is consistent with evidence that mental toughness varies by age, experience, achievement level, gender, and sport type (Nicholls et al., 2009). In the present synthesis, adult or college-age samples showed a significantly larger female-minus-male difference than minor or adolescent samples. One explanation is cumulative exposure: as athletes move into older and more demanding sport systems, organizational stressors such as training demands, selection pressure, performance evaluation, injury history, and role uncertainty become more salient (Arnold and Fletcher, 2012; Reardon et al., 2019). Any imbalance in recovery, social support, or institutional protection may therefore become more consequential when valued resources are threatened or depleted (Denckla et al., 2020). Competitive level showed a similar numerical direction, with more negative estimates in elite/professional and collegiate samples than in youth/developmental samples, but the formal moderator test did not support competitive level as a reliable between-subgroup difference. The developmental interpretation should therefore rest primarily on age stage, while competitive level should be treated as a related but currently unconfirmed pattern.

The team-sport pattern should also be interpreted cautiously. Team-sport samples showed a larger negative estimate than individual or mixed-sport samples, but the formal moderator test did not reach statistical significance, and the team-sport subgroup was small and highly heterogeneous. The pattern is nevertheless useful for hypothesis generation because team sports contain social mechanisms that may shape how resilience-related capacities are expressed, including role allocation, coach-athlete relationships, peer hierarchy, and access to playing opportunities. Newland et al. (2013) showed that mental toughness predicted basketball performance differently by gender and starter status, directly linking toughness to role opportunity. Gucciardi et al. (2017) found that mental toughness was associated with a weaker relation between controlling coaching and learning in elite adolescent netballers, and Karabulut and Akinci (2025) linked coach-athlete relational quality with mental toughness in adolescent female basketball players. Together, these studies suggest that team environments may shape the meaning of resilience-related capacities, but the present meta-analysis does not provide definitive evidence that sport type moderates female-male differences.

### Resilience-related capacity as a performance-health resource

The association synthesis showed that resilience-related capacities were moderately related to adaptive indicators in female athletes. This result is consistent with meta-analytic evidence linking resilience with mental health indicators (Hu et al., 2015), mental toughness with athletic performance (Hsieh et al., 2024), and mental toughness with broader psychological functioning across domains (Lin et al., 2017). The present study extends that evidence by showing that, within female-athlete samples, resilience-related capacities are not associated with only one domain. Positive associations were observed for performance or fitness, psychological well-being and resources, coping and adaptation, reduced distress or risk, and social or environmental resources.

The performance-related association supports the sport-specific value of resilience-related constructs. Athletic performance requires athletes to execute skills under fatigue, uncertainty, evaluative pressure, and rapid feedback, so confidence, persistence, attentional control, and challenge appraisal should be relevant to performance outcomes (Jones et al., 2002). Meta-analytic evidence also supports a positive association between mental toughness and athletic performance (Hsieh et al., 2024). In the present synthesis, the association with performance or fitness was similar in magnitude to the association with psychological well-being and resources. This result is important for women’s sport psychology because it links performance-related indicators with psychological sustainability without treating them as the same outcome domain. Female athletes’ resilience-related capacities were associated with performance or fitness indicators, but this association formed part of a broader adaptation profile rather than a purely competitive trait.

The psychosocial associations are especially relevant for interpreting female athletes’ functioning beyond performance outcomes. Resilience-related capacity was associated with psychological resources and with reduced distress or risk, which aligns with evidence that resilience, self-compassion, and social support are related to lower distress in women collegiate athletes during COVID-19 (Mikesell et al., 2023) and that resilience-related resources are linked to body satisfaction and disordered eating symptoms among Black women student-athletes (Dickens et al., 2023). These findings also fit the IOC consensus view that athlete mental health is shaped by individual, interpersonal, organizational, and sport-specific factors rather than by individual traits alone (Reardon et al., 2019). For female athletes, resilience-related capacity appears most closely aligned with more favorable psychosocial indicators when it is embedded in a resource system that supports emotion regulation, recovery, help-seeking, and a safer relationship with the athletic body.

However, the positive overall association should not be interpreted as evidence that more resilience is always better. Female-athlete studies show why the outcome domain matters. Madrigal et al. (2016b) found that mental toughness in female roller derby and rugby athletes was associated with approach coping and less adverse psychological response to injury, but also with sport-specific decisions about playing through injury. Codonhato et al. (2018) examined resilience in elite rhythmic gymnastics in a setting where stress, recovery, injury, scoring demands, and social support were tightly connected. Cooley A. P. et al. (2023) linked hardiness in Division I women’s lacrosse to perceived wellness and training responses, placing resilience-related traits in everyday training management rather than in abstract well-being alone. These studies suggest that resilience-related capacity is associated with coping and performance while also co-occurring with conditions that require recovery or structural change. Because most included studies were cross-sectional, these associations should not be interpreted as evidence that resilience-related capacities cause better psychosocial functioning, stronger performance, or lower distress. They instead indicate that resilience-related capacities co-occur with multiple indicators of female athletes’ adaptation.

### Implications

The findings have two related implications for resilience theory in sport. First, they support a conditional account rather than a simple adversity-builds-resilience account. Female athletes’ above-midpoint absolute scores are consistent with sport-training and sport-selection perspectives, because competitive sport repeatedly exposes athletes to challenge and may retain those who can function under pressure (Connaughton et al., 2008; Fletcher and Sarkar, 2012; Jones et al., 2002). At the same time, the small female-minus-male disadvantage, high heterogeneity, and moderator patterns are more consistent with dynamic and resource-based models, in which adaptation is more likely when protective systems are available and valued resources are not chronically depleted (Denckla et al., 2020; Hobfoll, 1989; Luthar et al., 2000; Masten, 2001). Second, the findings show that construct choice is not merely methodological, even though construct type was not confirmed as a statistical moderator of female-male differences. Psychological resilience and mental toughness showed similar absolute levels and both related positively to adaptive outcomes, while mental toughness showed a numerically more negative female-minus-male estimate. Measures emphasizing confidence, control, and pressure tolerance may therefore capture somewhat different aspects of gendered sport adaptation than measures emphasizing recovery or adaptation (Crust, 2007; Gucciardi et al., 2015; Lin et al., 2017; Smith et al., 2008). The theoretical contribution of this review is therefore to frame female-athlete resilience as a context-dependent performance-health adaptation, whose meaning depends on sport demands, gendered resources, developmental stage, measurement content, and the outcome used to define successful adaptation.

These conclusions also have practical implications for psychological support in women’s sport. Resilience interventions should not be reduced to persistence, emotional control, or pressure tolerance, even though those capacities can support performance. A narrow toughness model may discourage disclosure, recovery, and help-seeking in environments where athletes already receive strong messages to endure pain or hide distress (Bird and Earl, 2026; Madrigal et al., 2016b; Reardon et al., 2019). Programs for female athletes should therefore combine performance-relevant skills with recovery planning, stress appraisal, self-compassion, adaptive help-seeking, injury communication, body-related coping, and social support, consistent with female-athlete studies linking resilience with self-compassion, social support, sport confidence, training stress, and mental-skills development (Dickens et al., 2023; Gezer and Korucuk, 2026; Griffith et al., 2024; Mikesell et al., 2023; Wang et al., 2026). The small female-minus-male disadvantage also points to organizational responsibilities: coaching climate, psychological services, medical care, injury protection, anti-harassment procedures, nutrition and menstrual-health support, and the safety of seeking help without losing status or selection opportunities should be treated as part of the resilience system rather than as external background conditions (Mountjoy et al., 2018; Reardon et al., 2019).

### Limitations and future directions

Although this review synthesized 76 studies involving 27,386 coded participants, including at least 14,804 female athletes, its breadth also makes clear where the evidence remains uneven. Most included studies were cross-sectional, so the observed associations cannot establish whether resilience-related capacities are associated with later functioning, whether better functioning increases perceived resilience, or whether both reflect third variables such as coaching climate, social support, injury history, socioeconomic resources, or previous sport success. The decision to synthesize psychological resilience, mental toughness, hardiness, and closely related constructs allowed the review to capture the field as it is currently organized, but future studies should compare these constructs within the same female-athlete samples to test whether they predict different outcomes after accounting for shared variance. The review also benefited from a large gender-specific evidence base, yet many otherwise relevant studies did not report female-specific means, standard deviations, or correlations, which limited the association synthesis and some outcome-domain estimates. Finally, the available moderators, including sport type, age stage, and competitive level, were useful but broad proxies for more specific demands such as body evaluation, weight sensitivity, contact exposure, injury culture, coaching climate, funding, race, sexuality, disability, and national sport systems. Future work should use longitudinal and intervention designs, report gender-stratified statistics routinely, and measure stress exposure, resource access, and sport culture directly.

## Conclusion

Female athletes’ resilience-related scores were generally above the theoretical midpoints of the measures used, yet direct comparisons showed slightly lower scores than those of male athletes. Resilience-related capacity was also moderately associated with adaptive outcomes in female athletes, including both performance-relevant and health-relevant domains.

## Data Availability

Publicly available datasets were analyzed in this study. This data can be found here: https://osf.io/g3hfx/.
