# Supplementary material for: Resilience in female athletes: a meta-analysis of levels, gender differences, and associations across psychosocial, performance, and health domains
Source: Front Psychol. 2026 Sep 3;17:1948554. doi: 10.3389/fpsyg.2026.1948554 (PMC13581772; doi:10.3389/fpsyg.2026.1948554)
Supplement: Supplementary file 2 [file Supplementary_file_2.docx]

**Supplementary Appendix 1. Search strategies**

Searches were completed in July 2026. The strategy combined terms for resilience-related psychological capacities with terms for athletes and sport performers. Database-specific field tags and syntax were adapted to each platform. The search strings did not specify publication-year limits; English-language full text and empirical eligibility were assessed during full-text screening.

**Supplementary Table S1. Search strategies by database.**

| **Database** | **Records identified** | **Search strategy** | **Limits / filters** |
| --- | --- | --- | --- |
| Web of Science | 1,336 | TS=(("psychological resilien*" OR resilien* OR "sporting resilien*" OR "trait resilien*" OR hardiness OR "mental toughness") AND (athlet* OR "sport performer*" OR "professional sport*" OR "elite sport*" OR "elite athlete*" OR "student athlete*" OR "collegiate athlete*")) | No publication-year limit specified in the search string; English full text assessed during eligibility screening. |
| MEDLINE | 920 | ("psychological resilien*" OR resilien* OR "sporting resilien*" OR "trait resilien*" OR hardiness OR "mental toughness").ti,ab,kf. AND (athlet* OR "sport performer*" OR "professional sport*" OR "elite sport*" OR "elite athlete*" OR "student athlete*" OR "collegiate athlete*").ti,ab,kf. | No publication-year limit specified in the search string; English full text assessed during eligibility screening. |
| ProQuest | 495 | NOFT("psychological resilien*" OR resilien* OR "sporting resilien*" OR "trait resilien*" OR hardiness OR "mental toughness") AND NOFT(athlet* OR "sport performer*" OR "professional sport*" OR "elite sport*" OR "elite athlete*" OR "student athlete*" OR "collegiate athlete*") | No publication-year limit specified in the search string; English full text assessed during eligibility screening. |
| PubMed | 835 | ("psychological resilience"[Title/Abstract] OR resilien*[Title/Abstract] OR "sporting resilience"[Title/Abstract] OR "trait resilience"[Title/Abstract] OR hardiness[Title/Abstract] OR "mental toughness"[Title/Abstract]) AND (athlet*[Title/Abstract] OR "sport performer*"[Title/Abstract] OR "professional sport*"[Title/Abstract] OR "elite sport*"[Title/Abstract] OR "elite athlete*"[Title/Abstract] OR "student athlete*"[Title/Abstract] OR "collegiate athlete*"[Title/Abstract]) | No publication-year limit specified in the search string; English full text assessed during eligibility screening. |
| Scopus | 2,903 | TITLE-ABS-KEY("psychological resilien*" OR resilien* OR "sporting resilien*" OR "trait resilien*" OR hardiness OR "mental toughness") AND TITLE-ABS-KEY(athlet* OR "sport performer*" OR "professional sport*" OR "elite sport*" OR "elite athlete*" OR "student athlete*" OR "collegiate athlete*") | No publication-year limit specified in the search string; English full text assessed during eligibility screening. |
| PsycINFO | 739 | AB("psychological resilience" OR resilien* OR "sporting resilience" OR "trait resilience" OR hardiness OR "mental toughness") AND AB(athlet* OR "sport performer*" OR "professional sport*" OR "elite sport*" OR "elite athlete*" OR "student athlete*" OR "collegiate athlete*") | No publication-year limit specified in the search string; English full text assessed during eligibility screening. |
| IEEE Xplore | 44 | ("All Metadata":"psychological resilience" OR "All Metadata":resilien* OR "All Metadata":"sporting resilience" OR "All Metadata":"trait resilience" OR "All Metadata":hardiness OR "All Metadata":"mental toughness") AND ("All Metadata":athlet* OR "All Metadata":"sport performer" OR "All Metadata":"sport performers" OR "All Metadata":"professional sport" OR "All Metadata":"professional sports" OR "All Metadata":"elite sport" OR "All Metadata":"elite sports" OR "All Metadata":"elite athlete" OR "All Metadata":"elite athletes" OR "All Metadata":"student athlete" OR "All Metadata":"student athletes" OR "All Metadata":"collegiate athlete" OR "All Metadata":"collegiate athletes") | No publication-year limit specified in the search string; English full text assessed during eligibility screening. |

**Note.** Counts are the database-specific records identified before deduplication and screening, as reported in the manuscript search log. The total number of database records identified was 7,272.
