# Supplementary material for: Resilience in female athletes: a meta-analysis of levels, gender differences, and associations across psychosocial, performance, and health domains
Source: Front Psychol. 2026 Sep 3;17:1948554. doi: 10.3389/fpsyg.2026.1948554 (PMC13581772; doi:10.3389/fpsyg.2026.1948554)
Supplement: Supplementary file 3 [file Supplementary_file_3.docx]

**Supplementary Appendix 2. Forest plots**

**Supplementary Figure S1. Random-effects POMP summary forest plot for resilience-related capacities in female athletes.**

**Supplementary Figure S2. Random-effects forest plot for female-minus-male differences in resilience-related capacities.**
